# Probing Kinetic Mechanisms of Protein Function and Folding with Time-Resolved Natural and Magnetic Chiroptical Spectroscopies

**DOI:** 10.3390/ijms13010683

**Published:** 2012-01-10

**Authors:** David S. Kliger, Eefei Chen, Robert A. Goldbeck

**Affiliations:** Department of Chemistry and Biochemistry, University of California, Santa Cruz, CA 95064, USA; E-Mails: eechen@ucsc.edu (E.C.); goldbeck@ucsc.edu (R.A.G.)

**Keywords:** circular dichroism, optical rotatory dispersion, magnetic circular dichroism, magnetic optical rotatory dispersion, heme proteins, cytochrome, phytochrome, polarization, quasi-null, ligand shuttle

## Abstract

Recent and ongoing developments in time-resolved spectroscopy have made it possible to monitor circular dichroism, magnetic circular dichroism, optical rotatory dispersion, and magnetic optical rotatory dispersion with nanosecond time resolution. These techniques have been applied to determine structural changes associated with the function of several proteins as well as to determine the nature of early events in protein folding. These studies have required new approaches in triggering protein reactions as well as the development of time-resolved techniques for polarization spectroscopies with sufficient time resolution and sensitivity to probe protein structural changes.

## 1. Introduction

Circular dichroism (CD) spectroscopy has long been known as a valuable tool for determining the structural motifs of proteins. Like other structure-sensitive techniques, such as X-ray and NMR spectroscopies, in the past it had not been amenable to kinetic measurements and thus was useful only for determining static structural properties. In recent decades this changed, however, so that time-resolved CD measurements have become possible, thus opening up the capability of determining the time dependence of protein structural changes.

The first approach to time-resolved CD measurements involved the coupling of CD spectroscopy to stopped-flow kinetic techniques [1,2]. This approach used a standard technique for CD measurements in which the circular polarization of the light was alternated between left and right circular polarizations at frequencies on the order of 100 KHz. Stopped-flow techniques were capable of millisecond mixing times. The time course of CD signals was thus possible at single wavelengths with time resolution on the order of several milliseconds.

Efforts began in the mid 1980’s to improve the time resolution of CD measurements in order to follow structural changes occurring in proteins on faster time scales. To do that, a different approach to CD measurements was developed [3]. While oscillating between two circular polarizations with higher frequency is possible, it would have meant using smaller aperture optics, thus reducing light intensities to levels that would make the signal to noise ratio of CD measurements poor. Instead, a pseudo-null approach to CD measurements was developed which made CD measurements possible on a nanosecond time scale, 6 orders of magnitude faster than previous capabilities.

In this article we will describe how this pseudo-null technique works, how modifications of the technique can be used for magnetic circular dichroism (MCD), optical rotatory dispersion (ORD), and magnetic optical rotatory dispersion (MORD) measurements, and how these techniques have been used for rapid measurements to determine early events in protein function and folding reactions.

## 2. Quasi-Null Polarization Spectroscopy

**Figure 1:**
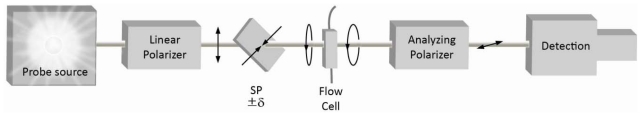
Apparatus for near-null ellipsometric time-resolved measurements of circular dichroism. Elliptically polarized light of high eccentricity is generated by sending initially unpolarized light through the first linear polarizer and then a strain plate (SP), which introduces a small phase retardance (δ). Right and left elliptically polarized light (REP and LEP) are obtained by rotating SP around its vertical axis by 180°. A circularly dichroic sample will change the eccentricity of elliptically polarized light because this light comprises components of LCP and RCP light with different amplitudes. After the sample, the LEP and REP probe light travel through a second, analyzing polarizer, and the minor axes (horizontally polarized light) of the two beams are detected by either an intensified photodiode array or intensified charge coupled device.

Typical CD signals represent differences in the absorption of left and right circularly polarized light that are on the order of 10^−3^ to 10^−5^ of the unpolarized absorption. Phase-sensitive detection approaches were thus used to detect the small differences in large signals but, as mentioned above, this limited the time resolution of measurements. To increase the time resolution of CD measurements significantly a different approach was employed in which the differences in intensity due to CD represented a sizeable fraction of the background signal. This involved taking advantage of the fact that elliptically polarized light can be thought of as a vector addition of left and right circularly polarized light components of different amplitudes. When elliptically polarized light passes through a circularly dichroic sample, the relative amplitudes of the left and right circular components change. This results in a change in the eccentricity of the elliptically polarized light. By measuring this change in eccentricity one can determine the circular dichroism with high sensitivity. The experimental arrangement to accomplish such a measurement is shown in Figure 1.

As seen in Figure 1, the light that probes CD signals (typically from a high intensity, short duration xenon flashlamp) passes through a linear polarizer to produce linearly polarized light. This light then passes through a strained quartz plate with the strain axis oriented at +45° or −45° to produce left or right elliptically polarized light. Enough strain is placed on the quartz plate to produce a retardation on the order of 1 or 2 degrees, producing elliptically polarized light with major axis intensity on the order of 10^4^ times the intensity of the minor axis. When this light passes through a sample exhibiting CD the polarization ellipticity is changed. The degree to which the ellipticity changes is monitored by passing the probe light through a second linear polarizer oriented to pass light aligned along the minor axis of the ellipse. This measurement monitors the intensity along the minor elliptical axis, an intensity that is changed substantially by the CD of typical samples. The light emerging from the second polarizer is directed to a detection system, typically a gated multichannel detector capable of monitoring an entire spectrum with several nanoseconds time resolution. Making such measurements with both left and right elliptically polarized light then yields the sample circular dichroism according to:

(1) $$\text{Signal}=(I_{\text{REP}}-I_{\text{LEP}})/(I_{\text{REP}}+I_{\text{LEP}})=2.3\;\Delta ɛcl/\delta$$

where *I*_REP_ and *I*_LEP_ are the intensities reaching the detector from right and left elliptically polarized light, respectively, Δɛ is the circular dichroism, *c* is the sample concentration, *l* is the sample path length, and δ is the retardation (in radians) of the strain plate producing the elliptically polarized light. Because of the high intensity of the probe beam and the amplification factor, δ, gained by the use of elliptically polarized light, it is possible to achieve nanosecond time resolution with this technique. With a nanosecond pulsed laser pump source, it is possible to trigger protein reactions (as discussed below) and follow the change in CD as a function of time from nanoseconds to seconds.

**Figure 2:**
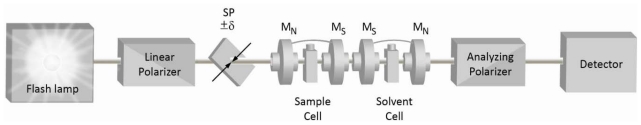
Apparatus for time-resolved measurements of magnetic circular dichroism. By addition of a magnet with the sample cell placed between the pole faces (M_N_, M_S_) and a compensator (solvent cell between pole faces of a second magnet), the TRCD approach can be extended to TRMCD measurements. The compensator counteracts the Faraday rotation of the probe beam polarization as it passes through the sample cell windows and the solvent in a magnetic field. It comprises a solvent blank in a cell matched with the sample cell and a magnetic field matched in magnitude but opposite in direction to that for the sample.

Modification of this approach makes it possible to measure magnetic circular dichroism, as shown in Figure 2 [4]. Here, the same basic optical layout is used, but the sample is placed in a magnetic field whose field lines through the sample are parallel to the probe light propagation direction. This magnetic field induces a circular dichroism, which can have significant magnitude in aromatic moieties. Of course, the field also produces an optical rotation (Faraday rotation) which can be quite large as it is due to rotation from the sample cell and solvent as well as the molecules under study. Since the quasi-null technique relies on measuring the intensity of light from the minor elliptical polarization axis, a rotation of the elliptically polarized light would yield a signal at the detector that is generally much larger than the signal due to the direct MCD effect. To counter this, the light is passed through a reference cell with solvent that is matched to the sample cell and which is placed in a magnet of the same intensity but opposite field orientation as the sample magnet [5]. This rotates the polarization ellipse back to the original orientation so that the light directed to the detector reflects the true CD signal of the sample.

The CD signal measured in this way is composed of both the natural CD of the sample as well as the magnetically-induced CD. In other words, the measured signal is CD + H•MCD, where H is the magnitude of the magnetic field oriented parallel to the probe propagation direction. If one is interested in determining the pure MCD signal, it is necessary only to reverse the magnetic field direction (so the field is oriented antiparallel to the probe propagation direction), thus yielding a signal of CD − H•MCD. The sum of these two measurements yields 2 CD and the difference of the measurements yields 2H•MCD.

With a slight modification, indeed a simplification, of the approaches shown in Figures 1 and 2 it is possible to measure ORD or MORD with high time resolution [6–8]. This involves removing the strain plate in the CD (MCD) apparatus and making measurements by rotating one of the polarizers by a small angle. This works in the following way. Starting with a sample between crossed polarizers, one of the polarizers is rotated by a small reference angle β (typically on the order of 1°). The light entering the second polarizer thus has its polarization altered by the rotation induced by the sample plus β. Rotating the first polarizer by −β results in the polarization entering the second polarizer changed by the rotation induced by the sample minus β.

(2) Signal=[I(β)-I(-β)]/[I(β)+I(-β)]≈-[LD+ORD]/β≈-ORD/β

Here LD is the sample linear dichroism, which will be negligibly small for a randomly oriented sample or for a photoselected sample that has been excited by a pump laser (propagating parallel to the probe beam propagation) whose polarization axis is parallel to the polarization axis of the fixed polarizer.

## 3. Kinetics of Protein Structural Changes

### 3.1. Protein Function

#### 3.1.1. Ligand Photolysis in Cytochrome c Oxidase

Since the development of the nanosecond time-resolved CD/ORD/MCD/MORD techniques described above they have been applied to the characterization of excited states of a number of inorganic metal complexes [9–12] as well as the study of tRNA [13] and a number of proteins. Proteins studied include heme proteins [7,14–24], phytochrome [25–29], PYP [30], Phot1-LOV2 [31], and rhodopsin [32]. As examples of the type of information obtainable from these time-resolved polarization spectroscopies, we will focus here on studies of bovine cytochrome *c* oxidase (cytox) and on phytochrome (phyt).

Cytox, the terminal enzyme in the respiratory chain of aerobic organisms, accepts electrons from cytochrome c. The reduced state of the protein binds molecular oxygen and reduces it to water. In this process, it creates a proton gradient across the mitochondrial membrane. This, in turn, drives the production of ATP within the mitochondria.

Bovine cytox is a complex, multi-subunit enzyme whose active site contains a heme *a*, with a nearby Cu_A_ and a heme *a*_3_, with a nearby Cu_B_. Heme *a* is a hexacoordinate heme A and it, with Cu_A_, are involved in accepting electrons from cytochrome *c*. The electron is then transferred to the ligand binding site, heme *a*_3_ and Cu_B_. Heme *a*_3_ is a pentacoordinate heme, with the fifth coordination site ligated to a histidine distal to the Cu_B_ and the sixth coordination site available to bind ligands such as O_2_, CO, or NO.

**Figure 3:**
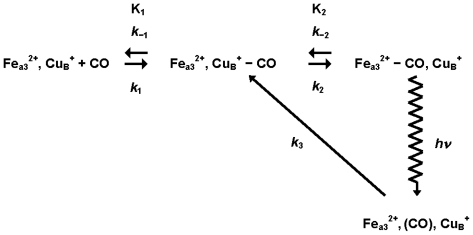
Mechanism of CO binding to cytochrome *c* oxidase deduced from time-resolved visible absorption, IR absorption, and Raman scattering experiments. Photolysis results in CO dissociating from heme *a*_3_ and rapidly binding to nearby Cu_B_. This mechanism would predict a high-spin state at the heme center for about a microsecond, at which point the high-field ligand CO would re-bind to heme a_3_ and return the heme center to a diamagnetic (*S* = 0) state.

In an effort to understand the mechanism of ligand reduction and proton transfer in cytox, early time-resolved studies used fast and ultrafast absorption spectroscopy, IR spectroscopy, and resonance Raman spectroscopy to study the reaction intermediates produced following photolysis of a CO ligand bound to heme *a*_3_ [33]. The results of those studies suggested that the CO rapidly binds to Cu_B_ after it is photodissociated from heme *a*_3_. The transfer of CO to Cu_B_ occurs on a picosecond time scale and time-resolved IR measurements showed that the CO remains on Cu_B_ for a couple of microseconds. Time-resolved resonance Raman measurements showed that upon photolysis of CO, a Raman peak indicative of an iron-histidine bond disappears, suggesting that the iron is in a low-spin state or in a 5-coordinate species where the 5th ligand is a group other than an imidazole. A mechanism consistent with all these measurements was proposed as shown in Figure 3.

**Figure 4:**
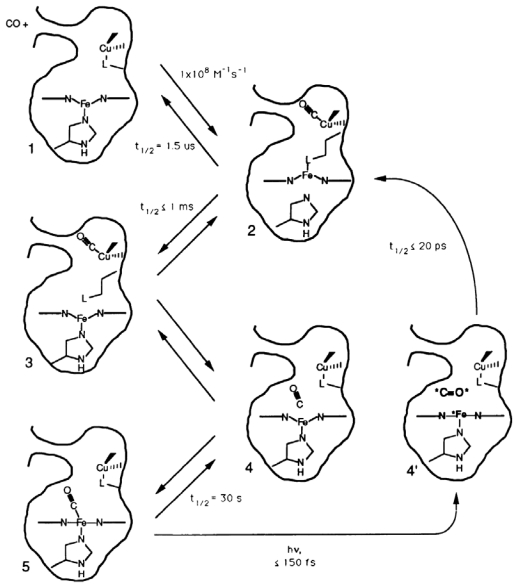
Cartoon of the Fe_a3_-Cu_B_ bimetal site of cytochrome *c* oxidase showing the ligand shuttle mechanism for CO binding, as deduced by combining the results of TRMCD spectroscopy with the data of earlier TROA, TRIR, and TR^3^ studies (the latter data considered alone having first suggested the mechanism shown in Figure 3). The combined data suggested shuttling of a ligand L between the heme *a*_3_ iron atom and Cu_B_, where L is a moiety endogenous to the protein. Species 1, 2, 3, 4, and 5 represent the thermal pathway. Species 4′ represents the photolytic generation of non-thermally equilibrated CO geminate to the heme *a*_3_ and Cu_B_ (asterisks indicate excess excitation energy imparted to CO and the heme). See text for a discussion of this mechanism.

That mechanism suggested that heme *a*_3_ should be high spin for a microsecond or so, until CO rebinds to it. To confirm this prediction time-resolved MCD measurements were made [16,19] because the MCD spectrum of fully reduced, unliganded cytox has a large contribution from high-spin heme a_3_ and a much smaller contribution from a low-spin heme. Interestingly, the TRMCD signal showed that a high spin species is created within 10 ns of photolysis of CO from heme *a*_3_ and the heme remains high spin for milliseconds, until CO rebinds to the heme. The apparent discrepancy between the Raman results and the MCD results was resolved with the proposal of a ligand shuttle model for cytox ligand binding, shown in Figure 4.

As Figure 4 shows, prior to photolysis, heme *a*_3_ is ligated to a histidine residue and to CO and Cu_B_ is ligated to another ligand, designated L. Upon photolysis of CO from heme *a*_3_ the CO group becomes ligated to Cu_B_ on a picosecond time scale, displacing L in the process. L then becomes ligated to heme a_3_ and, in the process, the histidine moiety dissociates from the heme. L can then rebind to Cu_B_ on a microsecond time scale, displacing CO in the process. The histidine rebinds to the heme, enabling CO to rebind to the 5 coordinate heme on a millisecond time scale.

This mechanism is rather unusual and would not have been proposed based on the absorption, IR, or Raman measurements alone nor based on the MCD measurements alone. However, the mechanism has strong support when data from all of the techniques are considered together. This points to the importance of applying multiple techniques to study complex biological problems.

#### 3.1.2. Light Activation and De-Activation of Phytochrome

Phytochrome (phyt) is the primary sensory pigment in green plants. It controls a wide variety of processes critical for the survival of plants, including seed germination, biosynthesis of chlorophylls, and flowering. At physiological temperatures, phyt can exist in two forms, an inactive form which absorbs red light and is thus referred to as Pr, and an active form which absorbs far-red light and is thus referred to as Pfr. These two forms can be interconverted by light, with absorption of red light by Pr producing Pfr and absorption of far-red light by Pfr producing Pr.

Time-resolved absorption experiments showed that the transformation from Pr to Pfr involves 5 intermediates with lifetimes in the microsecond and millisecond time scales [26]. The structural natures of these intermediates were probed by chromophore-band TRCD experiments discussed in [25]. The CD spectra of the later intermediates were similar to CD spectra obtained in studies where those intermediates were trapped at low temperatures. However, the earliest intermediate exhibited different CD spectra in the room temperature kinetic and low temperature trapping experiments. The difference in the CD spectra was interpreted as the result of structural strain in the low-temperature trapped intermediate, strain that relaxes within a microsecond at room temperature. This suggests that there is a structural change in the chromophore pocket at early times and, given that these differences are observable in CD spectra but not absorption spectra, that the early changes in the pocket involve motions of aromatic residues rather than charged groups.

To look at more global structural changes upon conversion of Pr to Pfr, TRCD measurements were carried out in the UV spectral region [29]. The results of this study showed that the major global structural change associated with the Pr to Pfr transition, an *N*-terminal α-helix folding, takes place in about 113 ms, significantly slower than the chromophore pocket structural changes observed at early times but faster than the last changes (slower than 266 ms) observed in absorption studies.

The photoreversion of Pfr to Pr was also studied by time-resolved absorption [27] and CD [28] studies. The absorption study showed that the reversion from Pfr to Pr reaction is simpler than the conversion reaction from Pr to Pfr in that it exhibits only 3 intermediates with lifetimes of 320 ns, 265 μs, and 5.5 ms in a sequential scheme. Interestingly, none of the intermediates involved in the Pfr to Pr reaction are the same as those observed in the Pr to Pfr reaction. As in the case of the Pr to Pfr reaction, TRCD measurements showed that the global unfolding process, involving a decrease in α-helix associated with the formation of the Pr form of the protein, does not occur in the last stage of the reaction, but is associated with the 265 μs process.

### 3.2. Protein Folding

#### 3.2.1. Rapid Folding Triggers

In order to study reaction kinetics it is necessary not only to be able to make measurements on a time scale comparable to the speed of the reaction, but also to be able to trigger the reaction on such a time scale. Several approaches have been used to trigger protein folding reactions. Early approaches to this problem involved mixing techniques [1,2]. Proteins in denaturing solutions would be mixed with solvents to dilute denaturants such that folding could occur. Alternately, proteins could be mixed with denaturant solutions to induce unfolding. This typically allowed for millisecond time resolution, though recent microfluidics techniques have allowed for sub-millisecond mixing times [34–36]. For faster folding, a variety of photo-triggering techniques have been used.

In special cases, the photochemical properties of proteins can be used to trigger folding. This has been done with considerable success in the case of folding of cytochrome *c* (cytc) (see below). Two trigger methods have been used for this protein. The first takes advantage of the fact that, while the heme in cytc is six coordinate in its native state, with the iron ligated to a histidine and a methionine group of the protein backbone, addition of CO to a partially denaturing solution of cytc leads to a displacement of the methionine ligation by CO, resulting in further destabilization of the protein. Thus, one can add enough denaturant to a solution of CO and cytc to unfold the ligated protein but not enough to denature the native protein. By photodissociating the CO from the heme with a fast laser pulse the CO-unbound protein will be formed rapidly under conditions that facilitate formation of the folded state [37]. The advantage of this approach is that CO photolysis reverses through ligand rebinding so the measurements can be repeated many times on the same sample. The disadvantage is that this reversibility means that only short-lived folding intermediates can be studied since CO rebinding kinetically competes with the folding process. An alternative folding trigger for cytc takes advantage of the fact that oxidized cytc is less stable than reduced cytc. Thus, denaturant can be added to an oxidized sample to unfold the protein at a concentration in which the reduced protein remains folded [38]. If a photoreducing agent, such as NADH, is added to the sample, photolysis of the NADH results in an electron transfer to the cytc. This rapidly produces reduced cytc in an environment in which it will fold to the native state.

Another approach to trigger folding is to insert a group which sterically hinders folding until a phototrigger alters the group to relieve the steric constraint. This approach has been used to trigger peptide folding using an azobenzene group attached to the peptide such that isomerization of the azobenzene can facilitate folding or unfolding of the peptide [39].

Finally, various perturbation approaches can be used to facilitate folding or unfolding of proteins. For example, dyes which produce a pH jump upon photoexcitation can be used to trigger folding or unfolding for pH-sensitive folding processes. Also, temperature jumps can be produced by tuning a pulsed laser to about 1.5 μm, where water absorbs, to induce unfolding reactions under heat denaturing conditions or folding for cold denatured proteins. Temperature jumps can cause significant birefringence artifacts for TRCD measurements so for measurements on time scales faster than birefringence relaxation times, temperature jumps should be coupled to TRORD measurements since birefringence artifacts affect ellipsometric CD measurements to first order but affect ellipsometric ORD measurements to second order [40].

#### 3.2.2. Monitoring Protein Folding Reactions

Perhaps the protein folding system most widely studied with ellipsometric polarization techniques is cytochrome *c*. TRCD, TRORD, and TRMCD have all been used to elucidate the early events in the folding mechanism of this protein. High time-resolution studies of cytc folding were first carried out using the CO photolysis approach to trigger folding and time-resolved absorption measurements to monitor folding [37]. TRCD experiments were later carried out to follow structural changes with more structure-sensitive far-UV CD measurements [41]. The first surprising result from this study was that absorption changes that were identified in the previous study as protein folding actually were due to changes in the ligation state of the heme. The second surprising result was that only about 7% of the native structure developed and that occurred within 500 ns of CO photolysis. Complete folding was prevented because of kinetic competition between the folding process and rebinding of the CO to the heme.

**Figure 5:**
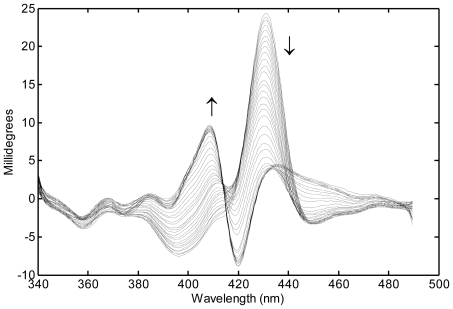
TRMCD spectra of cytochrome *c* measured at logarithmic time intervals from 330 ns to 25 ms after photolysis of the CO complex prepared under partially denaturing conditions. Arrows show direction of change with time. The spectra evolve through a complex mechanism, as revealed by the small blue shift over time of the ~430-nm MCD peak of the initial high-spin, 5-coordinate heme photolysis product. The mechanism proceeds first through unimolecular binding reactions between the heme iron atom and methionine or histidine residues, as the protein begins to fold, and finally through the bimolecular rebinding of CO, which stops refolding.

TRMCD studies of the cytc folding process revealed further interesting insights into the nature of folding. Folding had traditionally been described as a classical chemical reaction, with a reactant (unfolded protein) transforming into a product (folded protein) either directly or through some intermediate processes. An alternative model, the landscape view of folding, had been proposed [42] and supported by theoretical models. In this view, there are many unfolded protein states and each could, in principle, take different routes to the folded state. Thus, experimental observations monitored different folding routes with common observable features. The difference between these two views can be difficult to detect. If the rate of interconversion between different unfolded species (conformational diffusion) is fast relative to the rate of folding any observation will appear to monitor a classical reaction, *i.e.*, transformation from an average unfolded species to a folded species. On the other hand, if conformational diffusion times are comparable to folding times it would be possible to observe different folding pathways. TRMCD measurements of cytc folding, due to its rich spectral features (see Figure 5), were able to experimentally show, for the first time, that early events in the folding of cytc are better described by a landscape model than a classical model of folding [43]. Furthermore, by studying the folding in several cytc mutants the conformational diffusion time was determined to be about 3 μs [44], an order of magnitude faster than previous estimates for cytc based on absorption measurements [45]. Thus, folding processes occurring in microsecond times would best be described by a landscape view while millisecond or longer folding processes would adequately be described classically.

Beyond gaining understanding of the early events involved in cytc folding using the CO photolysis triggering approach, it is possible to monitor the whole folding process, from the earliest times to the formation of the native structure by using a photoreduction technique. This approach was first used in far UV-TRCD measurements [46]. Further evidence for the importance of the landscape view was demonstrated with this approach and an ultrafast folding process was observed which actually increased in amplitude with higher denaturant concentrations [47,48]. Interestingly, late folding processes leading to the native structure slowed down as expected as denaturant concentrations increased while early processes actually speeded up. This was shown to be related to a transient ligation to a non-native histidine group [49] associated with early formation of a molten globule folding intermediate [50].

## 4. Conclusions

From the examples cited above one can see that the ability to make CD, ORD, MCD, and MORD measurements on fast time scales is a valuable tool for biophysics. Two types of information of great value to understanding protein function are the structures of the proteins and the kinetics of protein reactions. The time-resolved polarization spectroscopies described here contribute to both of these areas and thus can provide important contributions to our mechanistic understanding of protein function. Furthermore, as our study of mechanistic features of cytox showed, different spectral techniques provide insights into different aspects of protein changes, so the ability to carry out protein studies with multiple spectroscopic probes is important for establishing mechanisms with confidence. The ability to link structural and kinetic information through these types of measurements has resulted in significant advances in our toolkit of biophysical methods for understanding proteins.

## References

[1] Anson M., Bayley P.M. (1974). Measurement of circular dichroism at millisecond time resolution: Stopped-flow circular dichroism system. J. Phys. E Sci. Instrum.

[2] Bayley P.M., Anson M. (1974). Stopped-flow circular dichroism: New fast-kinetic system. Biopolymers.

[3] Lewis J.W., Tilton R.F., Einterz C.M., Milder S.J., Kuntz I.D., Kliger D.S. (1985). New technique for measuring circular dichroism changes on a nanosecond time scale: Application to (carbonmonoxy) myoglobin and (carbonmonoxy)hemoglobin. J. Phys. Chem.

[4] Goldbeck R.A., Dawes T.D., Milder S.J., Lewis J.W., Kliger D.S. (1989). Measurement of magnetic circular dichroism (MCD) on a nanosecond timescale. Chem. Phys. Lett.

[5] Goldbeck R.A., Kliger D.S. (1993). Nanosecond time-resolved absorption and polarization dichroism spectroscopies. Methods Enzymol.

[6] Esquerra R.M., Goldbeck R.A., Kim-Shapiro D.B., Kliger D.S. (1998). Fast time-resolved magnetic optical rotatory dispersion measurements. 1. Mueller analysis of optical and photoselection-induced artifacts. J. Phys. Chem. A.

[7] Shapiro D.B., Goldbeck R.A., Che D., Esquerra R.M., Paquette S.J., Kliger D.S. (1995). Nanosecond optical rotatory dispersion spectroscopy: Application to photolyzed hemoglobin-CO kinetics. Biophys. J.

[8] Esquerra R.M., Goldbeck R.A., Kim-Shapiro D.B., Kliger D.S. (1998). Fast time-resolved magnetic optical rotatory dispersion measurements. 2. Confirmation of Mueller analysis and application to myoglobin ligand photolysis. J. Phys. Chem. A.

[9] Gold J.S., Milder S.J., Lewis J.W., Kliger D.S. (1985). Transient circular dichroism of the luminescent state of Ru(bpy)_3_^2+^. J. Am. Chem. Soc.

[10] Milder S.J., Gold J.S., Kliger D.S. (1990). Assignments of ground- and excited-state spectra from time-resolved absorption and circular dichroism measurements of the ^2^E State of (Δ)-Cr(bpy)_3_^3+^. Inorg. Chem.

[11] Milder S.J., Gold J.S., Kliger D.S. (1988). Time-resolved circular dichroism of the lowest excited state of (Δ)-Ru(bpy)_3_^2+^. Chem. Phys. Lett.

[12] Milder S.J., Gold J.S., Kliger D.S. (1986). Circular dichroism of a subnanosecond state: (Δ)-Fe(bpy)_3_^2+^. J. Am. Chem. Soc.

[13] Milder S.J., Weiss P.S., Kliger D.S. (1989). Time-resolved absorption, circular dichroism, and emission of transfer-RNA: Evidence that the photo-cross-linking of 4-thiouridine in transfer-RNA occurs from the triplet state. Biochemistry.

[14] Einterz C.M., Lewis J.W., Milder S.J., Kliger D.S. (1985). Birefringence effects in transient circular-dichroism measurements with applications to the photolysis of carbonmonoxyhemoglobin and carbonmonoxymyoglobin. J. Phys. Chem.

[15] Milder S.J., Bjorling S.C., Kuntz I.D., Kliger D.S. (1988). Time-resolved circular dichroism and absorption studies of the photolysis reaction of (carbonmonoxy)myoglobin. Biophys. J.

[16] Woodruff W.H., Einarsdottir O., Dyer R.B., Bagley K.A., Palmer G., Atherton S.J., Goldbeck R.A., Dawes T.D., Kliger D.S. (1991). Nature and functional implications of the cytochrome-*a*_3_ transients after photodissociation of CO-cytochrome oxidase. Proc. Natl. Acad. Sci. USA.

[17] Bjorling S.C., Goldbeck R.A., Paquette S.J., Milder S.J., Kliger D.S. (1996). Allosteric intermediates in hemoglobin. 1. Nanosecond time-resolved circular dichroism spectroscopy. Biochemistry.

[18] Chen E.F., Kliger D.S. (1996). Time-resolved near UV circular dichroism and absorption studies of carbonmonoxymyoglobin photolysis intermediates. Inorg. Chim. Acta.

[19] Goldbeck R.A., Dawes T.D., Einarsdottir O., Woodruff W.H., Kliger D.S. (1991). Time-resolved magnetic circular dichroism spectroscopy of photolyzed carbonmonoxy cytochrome *c* oxidase (cytochrome-*aa*_3_). Biophys. J.

[20] Goldbeck R.A., Einarsdottir O., Dawes T.D., Oconnor D.B., Surerus K.K., Fee J.A., Kliger D.S. (1992). Magnetic circular dichroism study of cytochrome *ba*_3_ from *Thermus thermophilus*: Spectral contributions from cytochrome *b* and cytochrome *a*_3_ and nanosecond spectroscopy of CO photodissociation intermediates. Biochemistry.

[21] O’Connor D.B., Goldbeck R.A., Hazzard J.H., Kliger D.S., Cusanovich M.A. (1993). Time-resolved absorption and magnetic circular dichroism spectroscopy of cytochrome *c*_3_ from *Desulfovibrio*. Biophys. J.

[22] Esquerra R.M., Goldbeck R.A., Kim-Shapiro D.B., Kliger D.S. (1998). Spectroscopic evidence for nanosecond protein relaxation after photodissociation of myoglobin-CO. Biochemistry.

[23] Goldbeck R.A., Esquerra R.M., Kliger D.S. (2002). Hydrogen bonding to Trp β37 is the first step in a compound pathway for hemoglobin allostery. J. Am. Chem. Soc.

[24] Vitale D.J., Goldbeck R.A., Kim-Shapiro D.B., Esquerra R.M., Parkhurst L.J., Kliger D.S. (2000). Near-ultraviolet magnetic circular dichroism spectroscopy of protein conformational states: Correlation of tryptophan band position and intensity with hemoglobin allostery. Biochemistry.

[25] Bjorling S.C., Zhang C.F., Farrens D.L., Song P.S., Kliger D.S. (1992). Time-resolved circular dichroism of native oat phytochrome photointermediates. J. Am. Chem. Soc.

[26] Zhang C.F., Farrens D.L., Bjorling S.C., Song P.S., Kliger D.S. (1992). Time-resolved absorption studies of native etiolated oat phytochrome. J. Am. Chem. Soc.

[27] Chen E.F., Lapko V.N., Lewis J.W., Song P.S., Kliger D.S. (1996). Mechanism of native oat phytochrome photoreversion: A time-resolved absorption investigation. Biochemistry.

[28] Chen E.F., Lapko V.N., Song P.S., Kliger D.S. (1997). Dynamics of the *N*-terminal α-helix unfolding in the photoreversion reaction of phytochrome A. Biochemistry.

[29] Chen E.F., Parker W., Lewis J.W., Song P.S., Kliger D.S. (1993). Time-resolved UV circular dichroism of phytochrome A: Folding of the *N*-terminal region. J. Am. Chem. Soc.

[30] Chen E.F., Gensch T., Gross A.B., Hendriks J., Hellingwerf K.J., Kliger D.S. (2003). Dynamics of protein and chromophore structural changes in the photocycle of photoactive yellow protein monitored by time-resolved optical rotatory dispersion. Biochemistry.

[31] Chen E.F., Swartz T.E., Bogomolni R.A., Kliger D.S. (2007). A LOV story: The signaling state of the Phot1 LOV2 photocycle involves chromophore-triggered protein structure relaxation, as probed by far-UV time-resolved optical rotatory dispersion spectroscopy. Biochemistry.

[32] Thomas Y.G., Szundi I., Lewis J.W., Kliger D.S. (2009). Microsecond time-resolved circular dichroism of rhodopsin photointermediates. Biochemistry.

[33] Woodruff W.H., Dyer R.B., Einarsdóttir Ó., Peterson K.A., Stoutland P.O., Bagley K.A., Palmer G., Schoonover J.R., Kliger D.S., Goldbeck R.A. (1991). Ultrafast and not-so fast dynamics of cytochrome oxidase: The ligand shuttle and its possible functional significance. P. Soc. Photo-Opt. Instrum. Eng.

[34] Akiyama S., Takahashi S., Ishimori K., Morishima I. (2000). Stepwise formation of α-helices during cytochrome *c* folding. Nat. Struct. Biol.

[35] Knight J.B., Vishwanath A., Brody J.P., Austin R.H. (1998). Hydrodynamic focusing on a silicon chip: Mixing nanoliters in microseconds. Phys. Rev. Lett.

[36] Hertzog D.E., Michalet X., Jager M., Kong X.X., Santiago J.G., Weiss S., Bakajin O. (2004). Femtomole mixer for microsecond kinetic studies of protein folding. Anal. Chem.

[37] Jones C.M., Henry E.R., Hu Y., Chan C.K., Luck S.D., Bhuyan A., Roder H., Hofrichter J., Eaton W.A. (1993). Fast events in protein folding initiated by nanosecond laser photolysis. Proc. Natl. Acad. Sci. USA.

[38] Pascher T., Chesick J.P., Winkler J.R., Gray H.B. (1996). Protein folding triggered by electron transfer. Science.

[39] Chen E.F., Kumita J.R., Woolley G.A., Kliger D.S. (2003). The kinetics of helix unfolding of an azobenzene cross-linked peptide probed by nanosecond time-resolved optical rotatory dispersion. J. Am. Chem. Soc.

[40] Chen E.F., Wen Y.X., Lewis J.W., Goldbeck R.A., Kliger D.S., Strauss C.E.M. (2005). Nanosecond laser temperature-jump optical rotatory dispersion: Application to early events in protein folding/ unfolding. Rev. Sci. Instrum.

[41] Chen E.F., Wood M.J., Fink A.L., Kliger D.S. (1998). Time-resolved circular dichroism studies of protein folding intermediates of cytochrome *c*. Biochemistry.

[42] Bryngelson J.D., Wolynes P.G. (1987). Spin glasses and the statistical mechanics of protein folding. Proc. Natl. Acad. Sci. USA.

[43] Goldbeck R.A., Thomas Y.G., Chen E.F., Esquerra R.M., Kliger D.S. (1999). Multiple pathways on a protein folding energy landscape: Kinetic evidence. Proc. Natl. Acad. Sci. USA.

[44] Abel C.J., Goldbeck R.A., Latypov R.F., Roder H., Kliger D.S. (2007). Conformational equilibration time of unfolded protein chains and the folding speed limit. Biochemistry.

[45] Hagen S.J., Hofrichter J., Szabo A., Eaton W.A. (1996). Diffusion-limited contact formation in unfolded cytochrome *c*: Estimating the maximum rate of protein folding. Proc. Natl. Acad. Sci. USA.

[46] Chen E.F., Wittung-Stafshede P., Kliger D.S. (1999). Far-UV time-resolved circular dichroism detection of electron-transfer-triggered cytochrome *c* folding. J. Am. Chem. Soc.

[47] Chen E.F., Goldbeck R.A., Kliger D.S. (2003). Earliest events in protein folding: Submicrosecond secondary structure formation in reduced cytochrome *c*. J. Phys. Chem. A.

[48] Chen E.F., Goldbeck R.A., Kliger D.S. (2004). The earliest events in protein folding: A structural requirement for ultrafast folding in cytochrome *c*. J. Am. Chem. Soc.

[49] Chen E., Abel C.J., Goldbeck R.A., Kliger D.S. (2007). Non-native heme-histidine ligation promotes microsecond time scale secondary structure formation in reduced horse heart cytochrome *c*. Biochemistry.

[50] Chen E., van Vranken V., Kliger D.S. (2008). The folding kinetics of the SDS-induced molten globule form of reduced cytochrome *c*. Biochemistry.

